# Mycosis fungoides bullosa: An unusual presentation of a rare entity

**DOI:** 10.1016/j.jdcr.2021.10.019

**Published:** 2021-10-20

**Authors:** Ahmad Nofal, Rania Alakad, Rana Ehab, Reham Essam

**Affiliations:** a Dermatology, Venereology and Andrology Department, Faculty of Medicine, Zagazig University, Zagazig, Egypt; b Interactive Dermatology Research Foundation, Cairo, Egypt

**Keywords:** bullous diseases, cutaneous T-cell lymphoma, mycosis fungoides, Ig, immunoglobulin, MF, mycosis fungoides

## Introduction

Mycosis fungoides (MF) is the most common type of cutaneous T-cell lymphoma with an incidence of 6 cases per million per year.^1^ Classic MF in adults can present in different stages, including the patch, plaque, and tumor stages.^2^ While patients with early patch or plaque stage MF usually have an indolent course, patients developing skin tumors require systemic or radiation therapy due to their aggressive course.^3^ In addition, several clinico-pathologic variants of MF have been described; eg, poikilodermatous, granulomatous, hypopigmented, folliculotropic, and vesiculobullous variants.^4^

Blistering is not usually associated with MF, but when present, it is typically associated with aggressive course and poor prognosis.^5^ Vesiculobullous MF has only been reported in 35 cases in the literature. Being a less recognized variant, it can easily be missed or confused with other bullous disorders, leading to delayed diagnosis and management.

We present a male patient with generalized vesiculobullous MF. The blisters were arranged in an annular pattern mimicking adult linear IgA bullous dermatosis. The lesions rapidly progressed to tumors necessitating aggressive treatment. The diagnosis of bullous MF was made based on the clinical and histologic findings.

## Case report

A 43-year-old man presented with a 1-year history of progressive, generalized pruritic skin eruption. There was no history of viral infections, contact allergy, systemic illness, or oral drug intake.

**Fig 1:**
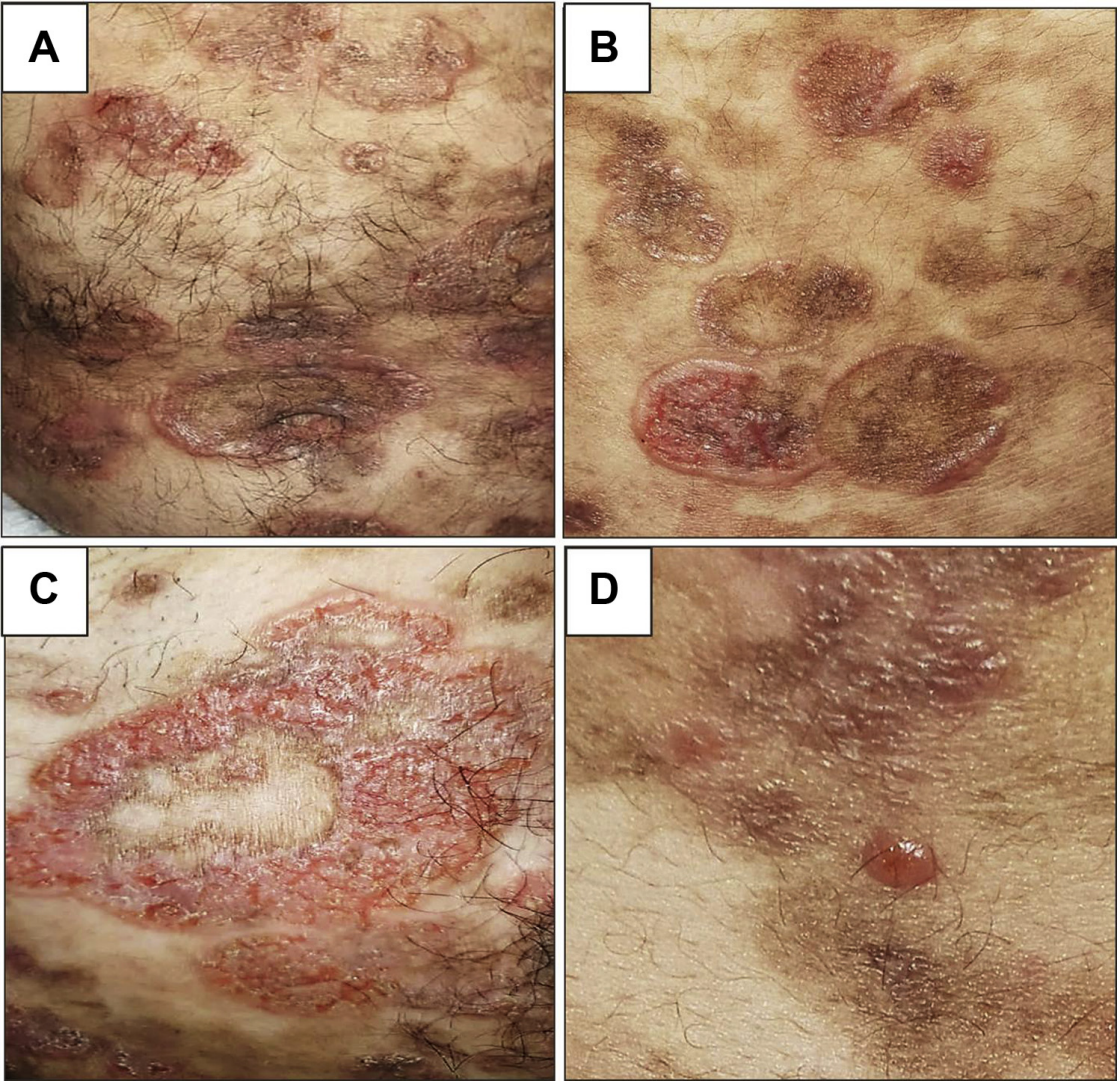
Vesiculobullous mycosis fungoides showing blisters arranged in an annular pattern on top of erythematous plaques (**A**, **B**); close-up view showing superficial erosions (**C**) and tense bulla (**D**).

Physical examination revealed generalized infiltrated plaques on the trunk and extremities covering most of his body surface area. The plaques were studded with multiple vesicles and bullae (flaccid and tense), arranged in a characteristic annular pattern mimicking adult linear IgA bullous dermatosis. Some of the blisters were ruptured and associated with exudative superficial erosions (Fig 1). There was no mucosal involvement. The patient was well-appearing and denied systemic symptoms.

**Fig 2:**
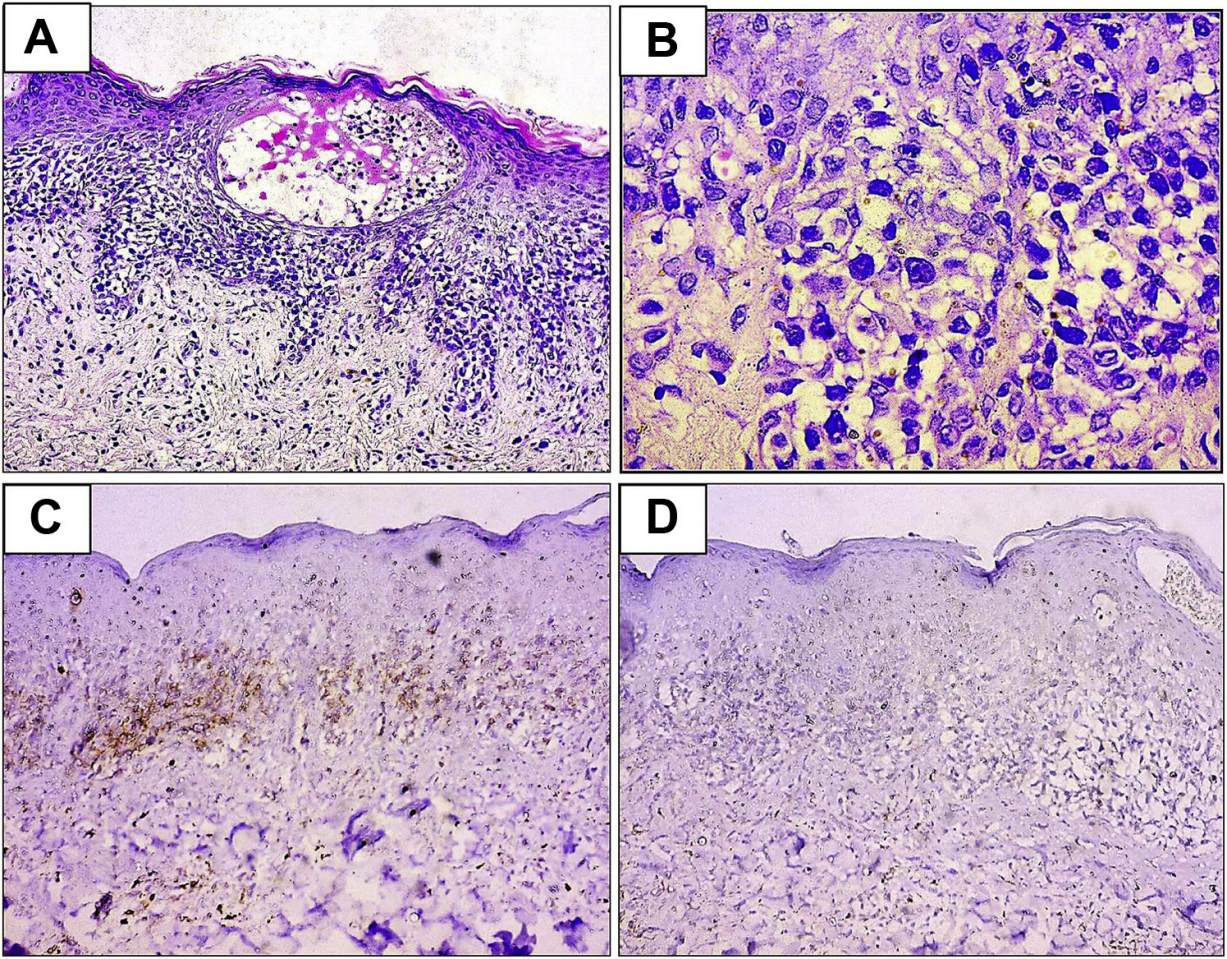
Histologic examination of vesiculobullous mycosis fungoides showing intraepidermal blistering, spongiosis, infiltration of epidermis by atypical lymphocytes. (Hematoxylin-eosin stain; original magnification: ×200.) (**A**), Atypical lymphocytes infiltrating the epidermis (epidermotropism) accompanied by epidermal spongiosis. (Hematoxylin-eosin stain; original magnification: ×400.) (**B**); immunohistochemical analysis showed positivity for CD4 (original magnification: ×200.) (**C**) and negativity for CD8 (original magnification: ×200.) (**D**).

Skin biopsies were taken from the plaques and bullae. Histologic examination revealed both intraepidermal and subepidermal blisters, along with infiltration of the upper dermis and dermo-epidermal junction by atypical lymphocytes with migration into the epidermis (epidermotropism) (Fig 2, *A*). With higher magnification, lymphoid cells were observed to be large with convoluted nuclei (Fig 2, *B*). Immunohistochemical analysis revealed the infiltrate to consist of predominantly T cells, the phenotype of which was CD3^+^, CD4^+^, and CD8^−^ (Fig 2, *C* and *D*). Direct immunofluorescence for IgG, IgA, immunoglobulin M, and C3 was negative.

**Fig 3:**
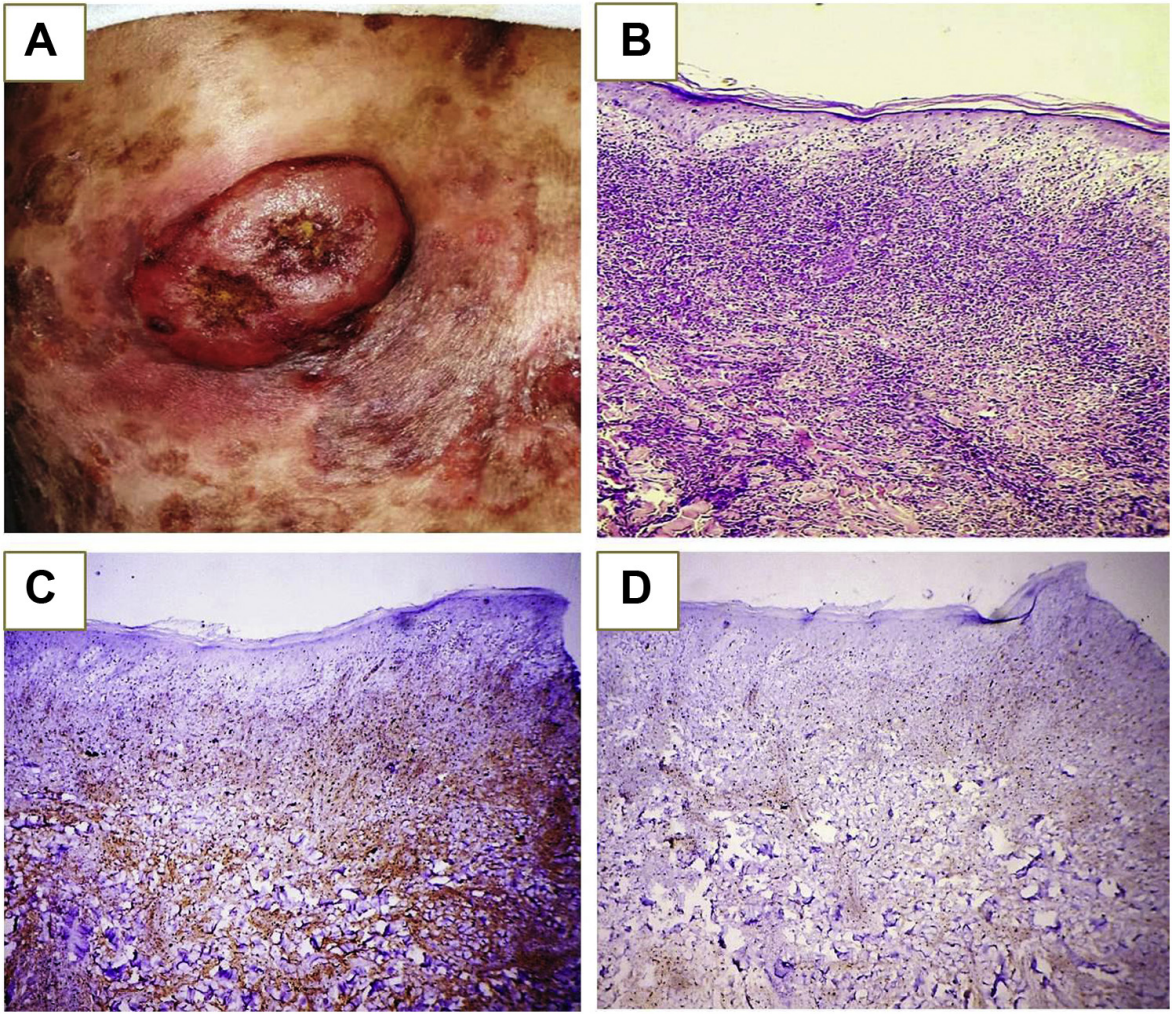
Erythematous nodule on the back with the ulcerated surface (**A**), histologic examination of the nodule showing an atypical lymphoid infiltrate involving the dermis and extending to the subcutaneous tissue with minimal epidermotropism (Hematoxylin-eosin stain; original magnification: ×200.) (**B**) Immunohistochemical stains of the biopsied tumor showed positivity for CD4 (original magnification: ×200) (**C**) and negativity for CD8 (original magnification: ×200.) (**D**).

Based on these findings, the diagnosis of vesiculobullous MF was made. The patient was lost to follow-up but returned 4 months later with new skin lesions on the back. Examination revealed a painless, erythematous, eroded 4 × 6-cm tumor on the back (Fig 3, *A*). The patient had a mobile, non-tender axillary lymph node (1 × 2 cm).

Histologic examination of the nodule showed diffuse atypical lymphoid infiltrate involving the full thickness of the dermis and extending to the subcutaneous tissue (Fig 3, *B*). By immunohistochemical analysis, the infiltrate was CD3^+^, CD4^+^, and CD8^−^ (Fig 3, *C* and *D*). Core biopsies of the axillary lymph node revealed reactive lymphadenopathy. A bone marrow biopsy revealed no involvement. Computed tomography of the neck, chest, abdomen, and pelvis was normal. Clinical and histologic findings were consistent with stage IIB MF (tumor stage). Despite aggressive therapy including cyclophosphamide, hydroxydaunorubicin, oncovin, and prednisolone, the condition rapidly progressed with continuous appearance of new lesions and tumors, resulting in death of the patient less than 1 year after the onset of the bullous lesions.

## Discussion

The vesiculobullous variant is a very rare clinical subtype of MF. According to Bowman et al,^6^ the diagnosis is made by the presence of the following: (1) Vesiculobullous lesions ± typical lesions of MF (patches, plaques, tumors); (2) typical histologic features of MF (atypical lymphoid cells, epidermotropism, Pautrier microabscess) with intraepidermal or subepidermal vesicles; (3) negative immunofluorescence to rule out autoimmune bullous diseases; and (4) negative evaluation for other possible causes of vesiculobullous lesions; eg, drugs, infections, porphyria.

The pathologic mechanism underlying blister formation in MF has not been elucidated. One explanation is the confluence of Pautrier microabscesses in MF lesions, which may lead to intraepidermal bulla formation.^7^ Alternatively, the proliferation of neoplastic lymphocytes and/or the release of lymphokines by malignant T cells may result in a loss of cohesion between keratinocytes and the basal lamina.^8^^,^^9^

The main differential diagnosis is autoimmune bullous disease, that has been reported to occur concurrently with classic MF.^10^^,^^11^ In our patient, the blisters were arranged in an annular pattern similar to adult linear IgA bullous dermatosis; however, immunofluorescence testing was negative.^12^ Annular MF has been previously described both in the absence of bullae^13^ and associated with vesicles and bullae, similarly to our case.^12^^,^^14^ Bullae in MF may also be seen in the setting of eczema herpeticum.^15^ Bullous drug eruption and bullous impetigo should also be considered as a differential diagnosis. In our case, this was excluded by the absence of history of drug intake and the absence of characteristic histologic findings; eg, necrotic keratinocytes and eosinophils.

The diagnosis of this rare entity is challenging, and the suspicion can only be raised by the presence of typical lesions of MF along with vesiculobullous lesions. Our patient had generalized MF plaques of prolonged duration with severe itching that developed into tumors in a short period of time.

This presentation highlights the aggressive nature of this variant. The treatment of bullous MF follows the usual treatment of MF according to the clinical stage. Phototherapy, methotrexate, interferon, bexarotene, radiotherapy, and histone deacetylase inhibitors are established treatments used in other variants of MF and have also been shown to improve bullous MF lesions.^16^^,^^17^

Epidemiologic, clinical, and histologic aspects of previously reported cases of bullous MF are presented in Table I. Review of previously reported cases revealed that bullous MF has a predilection for male patients with a mean age of 61.5 ± 15 years. The bullous lesions may arise *de novo* or on top of typical MF lesions. Bullous MF may present with flaccid or tense bullae, may have negative or positive Nikolsky sign, and may be generalized or localized, without specific predilection site. Regarding the histopathology of bullous MF, the line of cleavage may be intraepidermal, at different levels, or subepidermal; however, the majority of the patients displayed the CD4^+^ MF phenotype.

**Table I:** Summary of the reported cases of bullous mycosis fungoides

Reference	Clinical	Pathology	Associations
Kaposi, 1887^18^	Pemphigus-like lesions		
Lortat-Jacob and Legrain, 1926^19^	Few bullae with typical MF plaques	Intraepidermal and subepidermal bullae	
Goeckerman and Montgomery, 1931^20^	Generalized bullae with crusts	Subepidermal bullae	
O'Leary et al, 1935^21^	Few bullae on the abdomen		
Garb and Wise, 1943^22^	Blisters on the scalp, face, neck, and back Associated typical MF lesions	Intraepidermal and subepidermal bullae	Blisters mostly in the summer
Cawley et al, 1951^23^	Generalized vesicles and bullae	Atypical lymphocytes and lymphoblasts	
Roenigk and Castrovinci, 1971^11^	Generalized bullous eruption Nikolsky sign present	Large intraepidermal, subcorneal bullae with acantholytic cells	Alopecia mucinosa Pemphigus foliaceus
Kint et al, 1972^24^	Bullae on normal and involved skin		
Van Velde, 1974^25^	Bullae within MF plaques	Subepidermal bullae	
Konrad, 1982^8^	Bullae on both normal and affected legs		
Maeda et al, 1987^26^	Several bullous eruptions	Subcorneal bulla, numerous leukocytes, and large atypical lymphocytes	
Kartsonis et al, 1990^9^	Pruritic rash and blisters + MF plaques and tumor	Intraepidermal and subepidermal bullae	Follicular mucinosis Leonine facies
Turner et al, 1994^27^	Bullae on both normal and affected legs	Atypical lymphoid cells Subepidermal bullae	
Franken and Haneke, 1995^28^	Bullae on hands, legs, feet, and sole		
Aranha et al, 1997^29^	MF patches and plaques + vesicles and bullae	Subepidermal vesicle with infiltrate	Alopecia mucinosa Lymphadenopathy
McBride et al, 1998^30^	Extensive exudative lesions showing early blister formation	Dense lympho-histiocytic infiltrate in the dermis Blisters with epidermotropism	CD3^+^, CD4^−^, weakly CD8^+^
Ho et al, 2000^31^	Painful and large malodorous mass with bulla + intact bullae on chest and abdomen	Intraepidermal (foot) and subepidermal (abdominal wall) bulla formation	
Ono et al, 2004^32^	Bullae on the back and extremities Nikolsky sign +	Subepidermal blistering + atypical lymphocytes in the dermis	Inguinal lymphadenopathy Increased CD4/CD8 ratio Sézary cells
Gantcheva et al, 2005^33^	Plaque and tumors on head, trunk, and limb + vesicles and erosions	Intraepidermal blistering + epidermotropism	CD3^+^, CD4^+^, CD8^−^
Layegh et al, 2007^34^	Multiple bullous plaques		
Pearce et al, 2007^35^	Blistering on trunk and acral sites bullae on her fingers	Intraepidermal vesiculation with epidermotropism	Adenocarcinoma of the lung, colorectal carcinoma, and bladder carcinoma
Balighi et al, 2007^36^	Flaccid acral bullae on erythematous MF plaque and normal skin	Atypical lymphocytes Intraepidermal bulla	
Liu et al, 2008^37^	Vesicles on the lesion of the abdomen	Subepidermal bullae	CD4^+^
Kamran et al, 2008^38^	Large bullae on the limbs and trunk. Plaques with poikilodermic features	Subepidermal cleft with several atypical lymphocytes	Axillary lymphadenopathy
Kneitz et al, 2010^5^	Two erythematous plaques on thigh Intact bullae within plaques	Subcorneal and intraepidermal bullae	CD3^+^
Sato et al, 2011^39^	Plaques and tumors Acute bullous eruption	Subepidermal bullae	Leukemoid reaction CD4^+^, CD8^+^, CD30^−^
Xu et al, 2013^15^	MF patches and plaques, flaccid vesicles, bullae, and pustules Negative Nikolsky sign	Intraepidermal and subepidermal blisters	Inguinal and axillary lymphadenopathy Hepatosplenomegaly CD4^+^, CD8^−^, CD30^−^ IgG- and IgM+ for Herpes simplex virus -1
Korekawa et al, 2015^10^	Bullae and erythematous macules on trunk and extremities	Subepidermal bullae accompanied by extensive infiltrates of atypical lymphocytes	Bullous pemphigoid BP180 NC16A+ DIF: IgG and C3 deposits at dermoepidermal junction Eosinophilia CD4^+^, CD8^−^
Porntharukcharoen et al, 2017^12^	Multiple tense bullae	Marked epidermotropism and subepidermal vesicle	Large-cell transformed MF stage IV-B with lymphadenopathy CD4^−^, CD8^+^, CD30^+^
Ilhame et al, 2017^40^	Bullous erosions of the trunk and limbs Nikolsky sign negative	Epidermal cleavage + atypical lymphocytes	Inguinal lymphadenopathy CD4^+^, CD8^−^, CD30^−^
Juzot et al, 2020^16^	Blisters on the trunk and lower limbs	Subepidermal bullae	
Wu et al, 2020^41^	Flaccid bullae and erosions on the scalp, neck, trunk, and extremities	Intraepidermal and subepidermal bulla formation	Sézary syndrome CD4^+^,CD8^−^, CD30^−^
Nofal et al, 2021 (present case)	Erythematous annular plaques of MF Vesicles and erosions within infiltrated plaques	Subepidermal and intraepidermal blistering	CD4^+^, CD8^−^

*MF*, Mycosis fungoides.

The case is usually treated as plaque or tumor stage MF, taking into consideration that the appearance of bullous lesions in a patient with MF appears to carry a poor prognosis; almost half the reported patients died within 1 year of the appearance of bullae, despite aggressive therapy.^6^

To conclude, we present a case of vesiculobullous MF with a distinguished presentation and a rapidly progressive course. Dermatologists should keep in mind the diverse presentations of MF to avoid misdiagnosis and delayed management.

## Conflicts of interest

None disclosed.
